# Genetic Analysis of Avian Gyrovirus 2 Variant-Related *Gyrovirus* Detected in Farmed King Ratsnake (*Elaphe carinata*): The First Report from China

**DOI:** 10.3390/pathogens8040185

**Published:** 2019-10-12

**Authors:** Qianqian Wu, Xin Xu, Qinxi Chen, Jun Ji, Yunchao Kan, Lunguang Yao, Qingmei Xie

**Affiliations:** 1 Henan Provincial Engineering Laboratory of Insects Bio-reactor, Henan Provincial Engineering and Technology Center of Health Products for Livestock and Poultry, Nanyang Normal University, Nanyang 473061, China; qianqianwu929@126.com (Q.W.); chen7q9x10@126.com (Q.C.); kanyunchao@163.com (Y.K.); lunguangyao@163.com (L.Y.); 2 College of Animal Science, South China Agricultural University, Guangzhou 510642, China; xqm0906@126.com

**Keywords:** avian gyrovirus 2, variant-related gyrovirus, sequence analysis, phylogenetic analysis, amino acid sites

## Abstract

Avian gyrovirus 2 (AGV2), which is similar to chicken infectious anemia virus, is a new member of the genus *Gyrovirus*. AGV2 has been detected not only in chicken but also in human tissues and feces. This study analyzed 91 samples (8 from liver tissue and 83 from fecal samples) collected from king ratsnakes (*Elaphe carinata*) from 7 separate farms in Hubei and Henan, China, for AGV2 DNA using PCR. The results demonstrated a low positive rate of AGV2 (6.59%, 6/91) in the snakes, and all the positive samples were collected from the same farm. The AGV2 strain HB2018S1 was sequenced, and its 2376 nt genome comprised three partially overlapping open reading frames: VP1, VP2, and VP3. Phylogenetic analysis revealed that the HB2018S1 and NX1506-1 strains from chickens in China belong to the same clade and that they have a nucleotide identity as high as 99.5%. Additionally, recombination analysis showed that HB2018S1 might originate from the recombination of viruses similar to those detected in chickens and a ferret. A total of 10 amino acid mutation sites (44(R/K), 74(T/A), 256 (C/R), 279(L/Q), and 373(V/A) in AGV2 VP1; 60(I/T), 125(T/I), 213(D/N), and 215(L/S) in AGV2 VP2; and 83(H/Y) in AGV2 VP3) different from those observed in most reference strains were found in the genome of HB2018S1, indicating that the differences may be related to a transboundary movement among hosts, which needs further elucidation. To the best of our knowledge, this study is the first report on an AGV2-infected poikilotherm, suggesting that cross-host transmission of viruses with circular single-stranded DNA genomes would be a public health concern.

## 1. Introduction

Avian gyrovirus 2 (AGV2) belongs to the genus *Gyrovirus* and was originally classified in the family *Circoviridae* but was reassigned to the family *Anelloviridae* according to the recent viral taxonomy report of the International Committee on Taxonomy of Virus, which states that AGV2 is not structurally or genetically related to the members of the family *Circoviridae* [1]. Besides AGV2, the family *Anelloviridae* includes another recognized species—chicken anemia virus (CAV). CAV was commonly found in chickens worldwide, leading to significant losses in the poultry industry since it was first isolated from the bursa of chicken Fabricius in 1979 in Japan [2]. AGV2 was first detected in diseased chickens in Brazil in 2011, and it harbored approximately 40% nucleotide similarity with that of CAV [3]. The AGV2 genome is approximately 2.38 kb and a circular single-stranded DNA (ssDNA); it mainly comprises three overlapping open reading frames (ORFs) coding for VP1, VP2, and VP3 [4]. AGV2 infections in chickens can result in brain damage, mental retardation, and weight loss [5]. Although other specific symptoms of AGV2 infections have not been confirmed, autopsy-based studies have reported clinical manifestations such as hemorrhage, edema, glandular gastric erosion, and facial and head swelling in infected chickens [5]. Varela et al. (2014) used duplex quantitative real-time PCR assay to assess commercially available poultry vaccines and suggested that the widespread existence of AGV2 is associated with vaccine contamination [6]. AGV2 has also been reported in different regions of Europe, Latin America, Africa, South America, and Asia, indicating its global distribution [7,8,9,10]. In 2011, Sauvage et al. (2011) identified human gyrovirus, which shared 96% nucleotide identity with AGV2, from a skin swab of a healthy individual, indicating that AGV2 may infect humans as well [11]. Furthermore, AGV2 has been also detected in human blood samples [12]. In China, farmed king ratsnake is a highly distributed nontoxic snake species; moreover, snake meat has a high nutritional value and medicinal efficacy. Fortuitously, one farmed king ratsnake that died from bacterial infection was detected as AGV2-positive; therefore, the present study aimed to investigate AGV2 in farmed king ratsnakes in China and amplify its genome. We subsequently performed an in-depth sequence analysis on the basis of genetic evolution and amino acid mutations between the sequenced strain and reference strains.

## 2. Materials and Methods

### 2.1. Samples and Virus Detection 

In 2018, 91 samples (8 from liver tissues of snakes that died from bacterial infection and 83 from feces collected with individual sterile swabs and placed in sterile collection tubes) collected from 7 king ratsnakes from different farms (farms A–G) in Hubei and Henan, China, were tested for AGV2 by PCR using AGV2-specific primers (AGV2-F (5′-CGTGTCCGCCAGCAGAAAC-3′) and AGV2-R (5′-GGTAGAAGCCAAAGCGTCCAC-3′); from nucleotides 656–1001) on the basis of the highly conserved region of the AGV2 genome, as described previously [12]. The liver tissues were obtained via dissection to avoid contamination, and the liver tissue and fecal samples were washed with 1 mL phosphate-buffered saline, frozen and thawed thrice, immersed in liquid nitrogen, and ground into a homogenate. The homogenate was centrifuged at 5000 rpm for 10 min, and 0.2 mL of the supernatant was used for nucleic acid extraction. DNA and RNA were extracted using a DNA/RNA extraction kit (TransGen Biotech, Beijing, China) according to the manufacturer’s instructions. The extracted DNA was quantified using the NanoDrop ND-1000 (Thermo Scientific, Waltham, MA, USA) according to the manufacturer’s instructions using 1 µL of the DNA sample, and the quantified extracted DNA (concentration: >100 ng; ratios of 260:280: >1.8, and ratios of 260:230: >2.0) was stored at −20°C until use. 

### 2.2. Whole-Genome Sequencing of AGV2 

Whole-genome sequencing of AGV2 was performed using three pairs of overlapping primers designed by Yao et al. (2016), including primers for the first (1F: 5′-ATT TCCTAGCACTCAAAAACCCATT-3′ and 1R: 5′-TCTGGGCGTGCTCAATTCTGATT-3′; from nucleotides 1960–379), second (2F: 5′-TCACAGCCAATCAGAATT GAGCACG-3′ and 2R: 5′-TTCTACGCGCATATCGAAATTTACC-3′; from nucleotides 349–1082), and third (3F: 5′-TATTCCCGGAGGGGTAAATTTCGAT-3′ and 3R: 5′-CCCCTGTCCCCGTGATGGAATGTTT-3′; from nucleotides 1046–2027) fragments; the amplified fragments were 802, 733, and 981 bp in length, respectively. DNA was added to a mix comprising the reaction buffer, GC (guanine and cytosine) enhancer, 6 pmol upstream/downstream primers, 0.4 mM deoxynucleotide (dNTPs) solution (3 µL), and PrimerSTAR HotStart DNA polymerase (TaKaRa Biotechnology Co., Ltd., Dalian, China) to obtain a total reaction volume of 20 μL. Sequence amplification was performed under the following cycling conditions: initial denaturation at 98°C for 5 min followed by 30 cycles of denaturation at 98 °C for 10 s, annealing at 60 °C for 15 s, and extension at 72 °C for 10 s, with final extension at 72 °C for 10 min. The PCR products of the three fragments were cloned into a pMD18-T easy vector (TaKaRa Biotechnology Co., Ltd., Dalian, China) for future sequencing (Syn-Biotechnology, Suzhou, China). PCR and whole-genome sequencing were performed at least thrice. 

### 2.3. Recombination and Phylogenetic Analysis 

SeqMan (DNASTAR, Lasergene, Madison, Wisconsin) was used for contig-assembly of the partial sequences. The whole-genome sequence of the snake-originated strain was submitted to GenBank under the accession number MK840982. After alignment of the HB2018S1 and 25 AGV2 reference sequences downloaded from GenBank (detailed information of each reference strain is presented in Supplementary Materials Table S1) using Clustalx v1.83, phylogenetic trees were constructed on the basis of the nucleic acids of whole genomes using MEGA v.6.0 with the neighbor-joining method using the Kimura 2-parameter model, gap pairwise deletion, and bootstrap analysis of 1000 replicates [13]. Furthermore, the RDP 4.36 and SimPlot 3.5.1 software were used for the genetic recombination analysis of AGV2, and the assumed parental strain was used as the reference strain. RDP was executed with default parameter settings, which include the RDP, GENECONV, and Maxchi. The results obtained by RDP4 were confirmed using a boot-scanning method in the SimPlot program v.3.5.1 [14]. Meanwhile, phylogenetic trees were also constructed as described above on the basis of the genome region relative to the predicted recombination events. In addition, mutation sites were compared according to the amino acid sequences of the three ORFs.

## 3. Results

### 3.1. Positive Rates of AGV2 in the Snakes in China 

To increase our understanding of AGV2 infection in farmed king ratsnakes in China, 91 samples were collected and analyzed. The results indicated that five fecal samples (6.02%, 5/83) and only one liver tissue sample (12.5%, 1/8) were positive for AGV2. All six of these positive samples were collected from the same snake farm in Hubei, China (the detection results of AGV2 in this farm are presented in Supplementary Materials Figure S1). Detailed information on the snake samples positive for AGV2 is listed in Table 1. 

### 3.2. Whole-Genome Sequencing of AGV2 in Snakes

On the basis of the sequencing and contig-assembly results, the AGV2 strain identified in the liver tissues of king ratsnakes was named HB2018S1, and its genome comprised a 2376 nt long sequence. The sequencing results showed that the genomes of the six AGV2-positive snakes were 100% identical, indicating that they were the same strain. The nucleotide identity of HB2018S1 with the same clusters of the NX1506-1, NX1506-2, NX1510, and JL1508 reference strains was up to 99.5%. The lowest nucleotide identity of 95.3% was detected between HB2018S1 and S53/It from chickens. In addition, the nucleotide similarities between HB2018S1 and the three human strains 915F06007FD, CL33, and JQ690763 were 95.6%, 97.1%, and 96.8%, respectively.

### 3.3. Phylogenetic Analysis 

**Figure 1:**
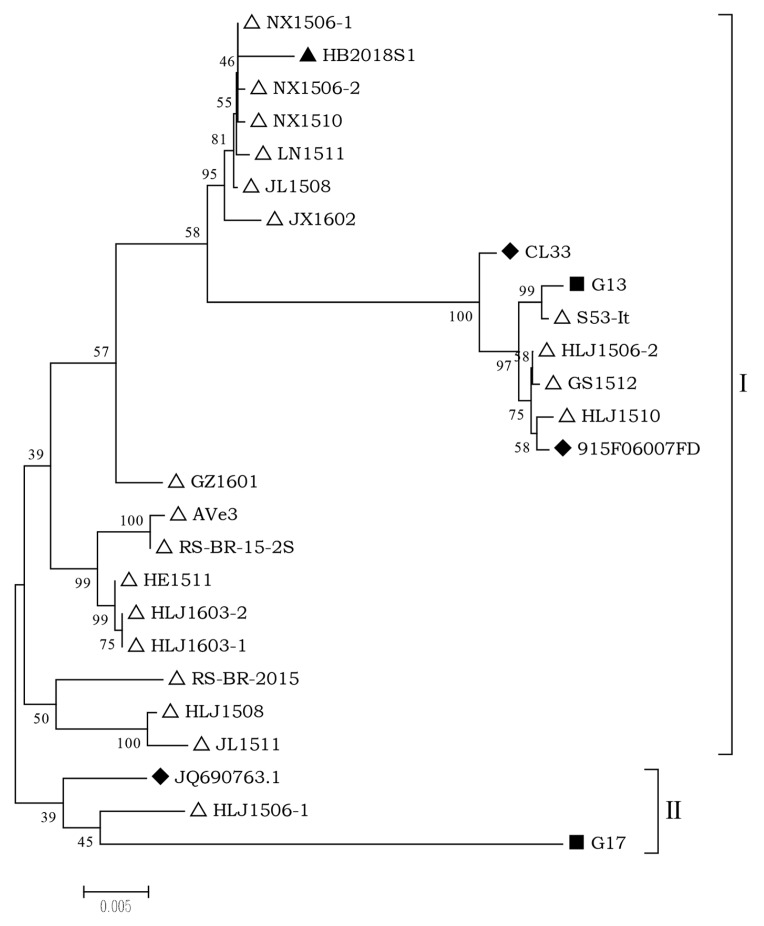
Phylogenetic analysis of the nucleotide sequences of HB2018S1 and 25 reference genomes available at GenBank. “△” indicates the virus identified in chickens; “◆” indicates the virus identified in humans; “■” indicates the virus identified in ferrets; “▲” indicates the AGV2 strain from snakes used in the present study.

Phylogenetic analysis revealed that the AGV2 and 25 full-length genome sequences downloaded from GenBank could generally be sub-grouped as two branches (Figure 1). HB2018S1 and most of the downloaded sequences belonged to branch I, which was subsequently divided into three clusters and marked. 

**Figure 2:**
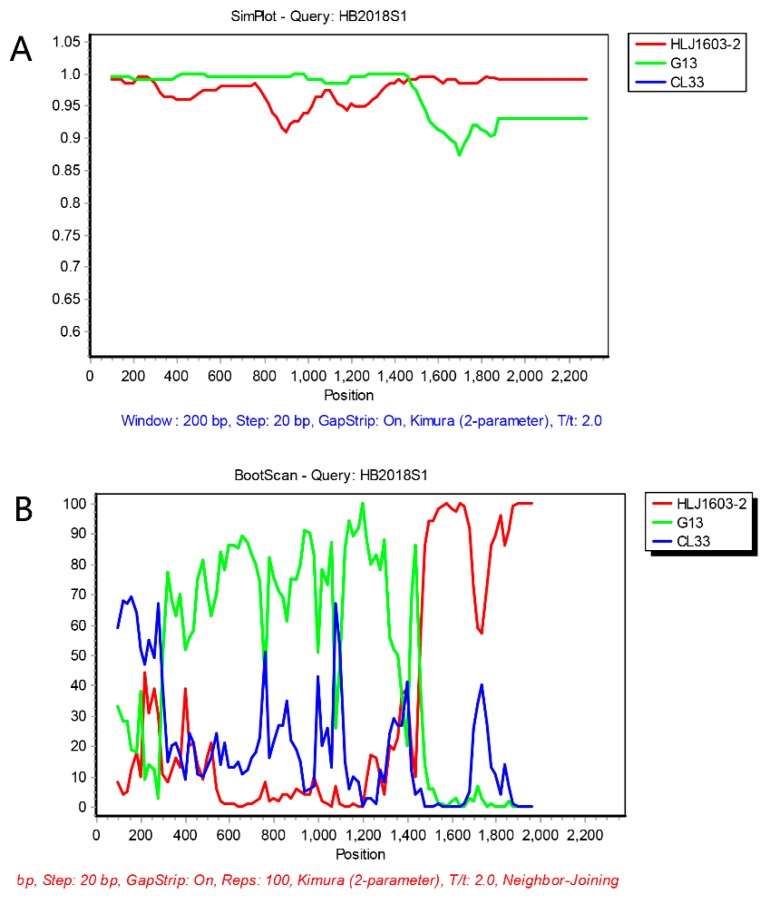
Simplot and BootScan analysis of the variant strain HB2018S1. HB2018S1 was a recombinant of the reference strains G13 and HLJ1603-2.

**Figure 3:**
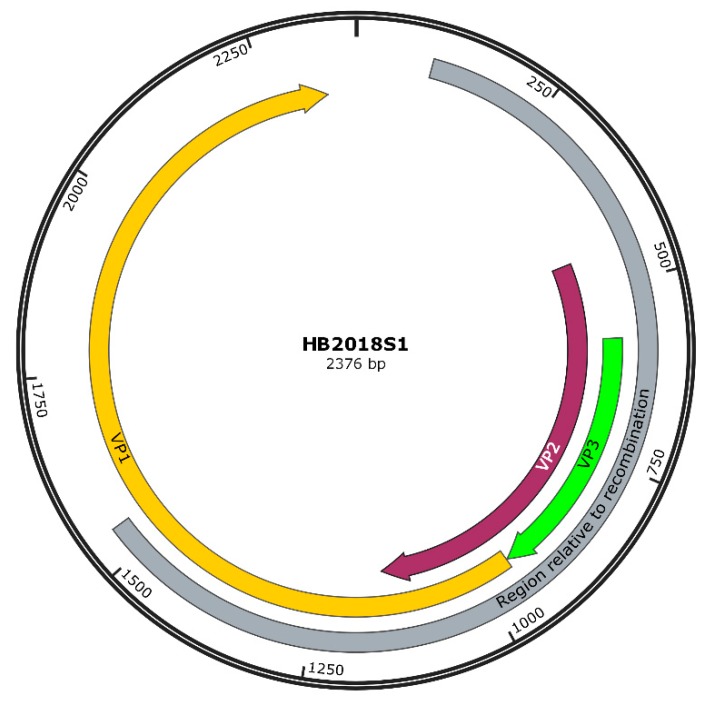
A map of the recombination event of HB2018S1 (accession no. MK840982) and its principal features. The principal features included VP1, VP2, and VP3, and the recombination is indicated in brown.

**Figure 4:**
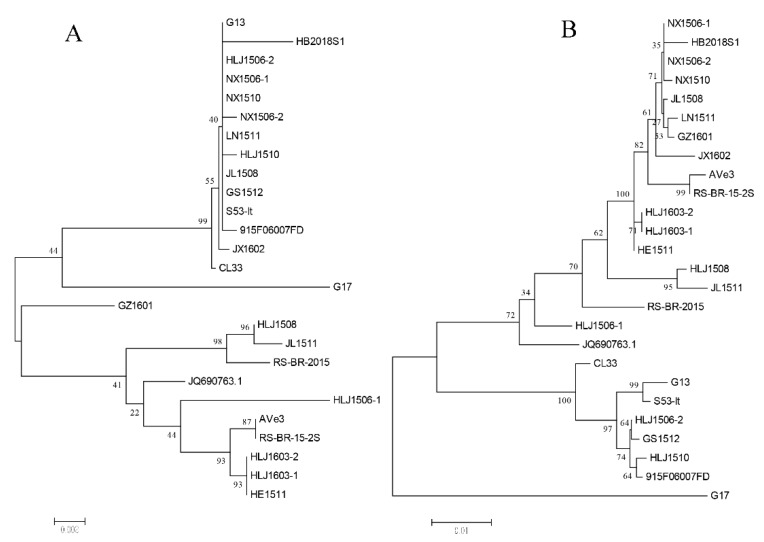
Phylogenetic trees of the partial genome regions between the recombination breakpoints. (**A**) 100–1542 nts of the genome nucleotides of all the strains; (**B**) 1543–2376 nts of the genome nucleotides of all the strains.

The SimPlot 3.5.1 and RDP 4.36 software were used to predict the possibility of recombination events in the AGV2 genome. As shown in Table 2, four recombination events were identified. In recombination event 1, the recombination sites of seven strains were identical, and all the strains covered belonged to branch I; moreover, in this study, HB2018S1 was also included in recombination event 1. The recombination occurred between the relatives of viruses within the clade of G13 (from CL33 to 915F06007FD) and the relatives of viruses within the clade of HLJ1603-2 (from Ave3 to HLJ1603-1). The recombinant virus gave origin to the whole clade that includes HB2018S1 (Figure 2). The recombination event was mapped at 100–1542 nts, and the region related to recombinant event is shown in Figure 3, which was created using the SnapGene software. This region (100–1542 nts) was similar to G13 (99.5%), and the remaining AGV2 genes were similar to HLJ1603-2 (99%). To further prove the occurrence of recombination events, we split the genome into two parts at recombinant sites 100 and 1542 nts and constructed phylogenetic trees. As shown in Figure 4A, according to the 100–1542 nts of the whole genome, HB2018S1 and G13 belong to the same branch and are far from HLJ1603-2. Conversely, phylogenetic trees constructed on the basis of the remaining partial whole genome show that HB2018S1 and HLJ1603-2 belong to the same branch and are far from G13 (Figure 4B). These results were in accordance with the recombination prediction.

### 3.4. Analysis of the Mutated Amino Acids 

The AGV2 genome comprises three overlapping ORFs, which encode VP1, VP2, and VP3 proteins. ORF3, which is located at sites 953–2335 and encodes VP1, comprises 460 amino acid residues. Three replication motifs were observed in the VP1 of HB2018S1: FAALS (325–329 residues), RRWLTLV (363–369 residues), and KAMA (412–415 residues). VP1 of HB2018S1 was 97.4–98.9% identical with the reference strains in terms of deduced amino acids. On the basis of the sequence alignment between HB2018S1 and the reference strains, 20 amino acid mutations were observed for VP1 (displayed in gray in Table 3), and VP1 of HB2018S1 had five unique amino acid mutations at sites 44(R/K), 74(T/A), 256(C/R), 279(L/Q), and 373(V/A). 

The ORF1 encoding AGV2 VP2 was located at sites 450–1145 and encoded a 231-amino acid long protein sequence, and the VP2 sequences of HB2018S1 were 94.0%–98.3% identical to those of the reference strains in terms of deduced amino acids. Sequence alignment indicated 15 hyper-mutated amino acid sites located in the AGV2 VP2 of HB2018S1 and reference sequences, whereas G17 from ferrets had an S insertion at site 162. In addition, the substitutions 60I/T, 125T/I, 213D/N, and 215L/S occurred only in HB2018S1 (displayed in gray in Table 4).

ORF2 encodes VP3, a nonstructural protein comprising 124 amino acid residues. HB2018S1 VP3 was 92.0%–99.2% identical to the other strains. Compared with the amino acid substitution sites of the majority of the reference strains, HB2018S1 only had an 83H/Y amino acid substitution, excluding G17, which had an R insertion at site 122 (displayed in gray in Table 5). 

The untranslated region (UTR) of HB2018S1 was located between the canonical polyadenylation site (AATAAA) and the start site of transcription, which included six closely matching direct repetition (DR) regions with a length of 22 nts. Among these, five DRs were identical (5′-GTACAGGGGGGTACGTCACCAT-3′). The different DR was the last DR. The three bases at its 3′ end were “A, G, and C” of HB2018S1, which were similar to the reference strains.

## 4. Discussion

To the best of our knowledge, AGV2 has been detected in three different hosts—chickens, humans, and ferrets [10,11,12]. Most AGV2 strains are mainly detected in chickens, which is the original host. In the present study, AGV2 was detected, for the first time, in cold-blooded farmed king ratsnakes; however, how the snakes were infected with the virus remains unknown. Farmed snakes are usually given a specific feed and occasionally raw meat. In this study, snakes from only one farm were positive for AGV2. According to a survey regarding the feed, the infected snakes (all from the same farm) had been fed chicken meat 1 month before sampling, whereas those in the other farms had been fed chicken meat supplied from different chicken farms within or after 1 week. On the basis of a random investigation of all the poultry farms that supplied chicken meat, the positive ratio for AGV2 of each farm reached 40%. It is supposed that the AGV2 detected in snakes originated from infected chickens, and that HB2018S1 might harbor the capability of cross-host transmission for only one farm whose snakes were tested to be AGV2-positive. Although it is challenging to prove that the origin of the AGV2 detected in the snakes was the chicken meat used as feed because the virus was not successfully isolated from chicken meat, AGV2 detected in snake liver tissues may actually indicate that the virus replicates in the host. Furthermore, the positive liver tissue and fecal samples collected from the same farm suggested that the fecal samples positive for AGV2 did not correspond to residual viruses from the gut because the snakes from other farms negative for AGV2 may also have been fed infected chickens, given that the AGV2 infection rate in chickens is very high in China [5,12]. Whether snakes are infected with AGV2 because of alimentary transmission following chicken consumption requires further investigation. In 2012, AGV2 was detected in the feather sacs of two adjacent chicken flocks in southwestern Brazil, suggesting a horizontal transmission mode of AGV2; however, more studies are required to demonstrate this phenomenon. From 2011 to 2013, some researchers conducted an epidemiological analysis of AGV2 in the serum and feces of some individuals to determine whether AGV2 could be replicated in humans. Whether the human AGV2 is attributable to metabolic residues in poultry food remains to be clarified. Phylogenetic analysis showed that the genome sequence of HB2018S1 was highly similar to those of NX1506-1, NX1506-2, and NX1510 from China. However, the local regions, wherein these strains were identified, are far from the collection site of HB2018S1, and the genome sequences of some strains from the chicken farms that supplied the chicken meat were distant from HB2018S1 (data not shown). Therefore, the AGV2 transmission mode requires further investigation. 

The AGV2 genome is 2376 nt long, similar to the AGV2 genome from chickens in China, having three overlapping ORFs, similar to those in chicken infectious anemia virus (CIAV) encoding VP1, VP2, and VP3. At the protein/amino acid level, the similarity of the VP1, VP2, and VP3 proteins in AGV2 to the VP1, VP2, and VP3 proteins in CIAV was 38.8%, 40.3%, and 32.2%, respectively [3]. The UTR, which is located between the polyadenylation site and the site of transcription initiation, is similar to CIAV in structure [15,16].

Previous reports have indicated that the AGV2 infection rate in chickens increases with time [5,12]. If AGV2 was transmitted from chickens to snakes in this study, the most probable explanation for only one virus strain or virus originating from one strain being detected and sequenced in snakes was the mutation of functional genes, resulting in trans-host infection. The VP1 protein of CIAV is the only capsid protein contained in the virus, and constitutes the antigen that elicits the appearance of neutralizing antibodies. There are three replication motifs on the VP1 sequence in CIAV, including FATLT (at 313–317 residues), QRWHTLV (at 351–357 residues), and YALK (at 402–405 residues) located on the VP1 sequence conserved in CIAV [17]. Similar sequences were observed in the VP1 of HB2018S1, and the sites are completely conserved in the currently reported AGV2 sequences. On the basis of part of the AGV2 sequences, Yao et al. (2016) hypothesized that a high-variant region exists in the middle and back of the VP1 protein, ranging from 288 to 314 residues. In the present study, only eight strains of viruses, including GS1512, HLJ1508, G13, JL1511, HM590588, JQ690763, RS/BR/15/25, and G17, had mutations, and the mutation sites were not identical. Because of the limited number of sequences, it was not possible to predict the high-variation regions accurately. However, further studies are required to verify whether the five mutations in the VP1 protein in HB2018S1, which are different from those in the other sequences, may affect viral host selectivity.

The VP2 protein in CIAV is a dual-specific protein phosphatase [18]. The WX7HX3CXCX5H sequence of the phosphatase motif is also highly conserved in CAV, torque teno virus (TTV), and TTV-like mini virus (TLMV) [17,19]. A similar motif in the VP2 protein of AGV2 are WLRQCARSHDEICTCGRWRSH (95–115) residues. Site-directed mutagenesis of VP2 revealed that VP2 affects the replication of viral particles, resulting in cytopathological changes and downregulating the levels of major histocompatibility complex I (MHC I) in infected cells [18]. VP1 and VP2 are necessary for viral particle replication. Noteborn [20] also observed interactions between VP1 and VP2 proteins on the basis of an immunoprecipitation test, which further confirmed that the nonstructural protein VP2 plays a scaffold role in virus particle assembly. In the VP2 protein, HB2018S1 has four different amino acid mutations and an S insertion site of G17 from ferrets. Further studies are warranted to determine whether such mutations alter VP2 function and, in turn, affect virus replication, and whether they affect the scaffold function of the VP2 protein.

The VP3 protein in CIAV is also known as apoptin. In normal cells, the VP3 protein exists in the cytoplasm. However, in cancerous cells, it exists in the nucleus and induces apoptosis [21,22]. It has also been demonstrated that the VP3 protein in AGV2 induces specific apoptotic functions in tumor cells, whereas the change in molecular function caused by a mutation at site 83H/Y of the VP3 protein in HB2018S1 still requires further experimental evidence.

In the present study, in addition to the AGV2 identified from chickens, humans, and ferrets, an AGV2 strain from a cold-blooded farmed snake was identified, which could provide insights into the modes of transmission of AGV2 and its host diversity.

## Figures and Tables

**Table 1:** Detailed information regarding the snake samples used in this study.

Farm	Province	Sample Quantity	Positive Rates	
			Fecal Sample	Liver Tissue Sample
A	Henan	10	0/9	0/1
B	Hubei	13	5/11	1/2
C	Hubei	5	0/5	0/0
D	Hubei	21	0/20	0/1
E	Henan	19	0/17	0/2
F	Hubei	11	0/10	0/1
G	Hubei	12	0/11	0/1
Total		91	5/83	1/8

**Table 2:** RDP 4.36 software recombination analysis of the avian gyrovirus 2 (AGV2) genome.

Recombination Event	No.	Recombinant Sequence	Breakpoint Positions		Major Parent		Minor Parent		*p*-Value
			Begin End		Similarity		Similarity		
**1**	7	HB2018S1	100	1542	HLJ1603-2	99%	G13	99.5%	2.71 × 10^−11^
**2**	1	HLJ1506-1	1948	2556	GZ1601	99.3%	G17	99.8%	4.50 × 10^−7^
**3**	1	GZ1601	1110	1542	JL1511	97.3%	S53-It	100%	3.38 × 10^−6^
**4**	3	JL1511	52	639	JQ690763.1	98.8%	G13	99.3%	1.89 × 10^−4^

Recombination event 1: NX1510; NX1506-2; NX1506-1; JL1508; JX1602; LN1511. Recombination event 4: HLJ1508; RS-BR-2015. Minor parent = parent contributing the smaller fraction of sequence. Major parent = parent contributing the larger fraction of sequence. *p*-value: *p*-value of the RDP method.

**Table 3:** Mutations at different amino acid sites in AGV2 VP1.

Strains	Substitution of Amino Acid Residues																			
	36	44	74	95	154	212	242	256	270	279	288	293	310	311	314	373	383	401	416	459
**HB2018S1**	G	K ^a^	A	K	A	K	R	R	S	Q	V	G	E	D	R	A	P	V	L	N
**NX1510**	G	R	T	K	A	K	R	C	S	L	V	G	E	D	R	V	P	V	L	N
**NX1506-2**	G	R	T	K	A	K	R	C	S	L	V	G	E	D	R	V	P	V	L	N
**NX1506-1**	G	R	T	K	A	K	R	C	S	L	V	G	E	D	R	V	P	V	L	T
**JL1508**	G	R	T	K	A	K	R	C	S	L	V	G	E	D	R	V	P	V	L	N
**LN1511**	G	R	T	K	A	K	R	C	S	L	V	G	E	D	R	V	P	V	L	T
**JX1602**	G	R	T	K	A	K	R	C	S	L	V	G	E	D	R	V	P	V	L	N
**GZ1601**	S	R	T	K	A	K	R	C	S	L	V	G	E	D	R	V	P	V	L	N
**HLJ1603-2**	G	R	T	K	A	K	R	C	S	L	V	G	E	D	R	V	P	V	L	N
**HLJ1603-1**	G	R	T	K	A	K	R	C	S	L	V	G	E	D	R	V	P	V	L	T
**HE1511**	G	R	T	K	A	K	R	C	S	L	V	G	E	D	R	V	P	V	L	T
**HLJ1506-2**	G	R	T	K	A	K	R	C	S	L	V	G	E	D	R	V	P	V	L	N
**GS1512**	G	R	T	K	A	K	R	C	S	L	V	G	E	D	K	V	P	V	L	N
**HLJ1508**	G	R	T	T	S	K	R	C	S	L	Q	Q	E	D	R	V	P	V	L	N
**G13**	G	R	T	K	A	K	R	C	S	L	V	G	E	E	R	V	P	V	L	N
**JL1511**	G	R	T	T	S	K	R	C	S	L	Q	Q	E	D	R	V	P	V	L	N
**HM590588**	G	R	T	T	S	R	G	C	A	L	V	G	Q	D	R	V	Q	M	I	N
**JQ690763**	G	R	T	K	A	K	R	C	S	L	Q	Q	E	D	R	V	P	V	L	N
**RS/BR/15/2S**	G	R	T	K	S	K	R	C	A	L	V	G	Q	D	R	V	Q	M	I	N
**HLJ1506-1**	S	R	T	K	A	K	R	C	S	L	V	G	E	D	R	V	P	V	L	N
**915F06007FD**	G	R	T	K	A	K	R	C	S	L	V	G	E	D	R	V	P	V	L	N
**G17**	S	R	T	K	S	K	R	C	S	L	Q	Q	E	D	K	V	P	V	L	N
**CL33**	G	R	T	K	A	K	R	C	S	L	V	G	E	D	R	V	P	V	L	N
**S53/It**	G	R	T	K	A	K	R	C	S	L	V	G	E	D	R	V	P	V	L	N
**HLJ1510**	G	R	T	K	A	K	R	C	S	L	V	G	E	D	R	V	P	V	L	N

^a^ Mutations were displayed in gray.

**Table 4:** Mutations at different amino acid sites in AGV2 VP2.

Strains	Substitution of Amino Acid Residues												
	14	60	125	141	156–158	161	162	165	167	174–175	179	213	215
**HB2018S1**	N	T ^a^	I	R	GKR	Y	-	A	T	EE	A	N	S
**NX1510**	N	I	T	R	GKR	Y	-	A	T	EE	A	D	L
**NX1506-2**	N	I	T	R	GKR	Y	-	A	T	EE	A	D	L
**NX1506-1**	N	I	T	R	GKR	Y	-	A	T	EE	A	D	L
**JL1508**	N	I	T	R	GKR	Y	-	A	T	EE	A	D	L
**LN1511**	N	I	T	R	GKR	Y	-	A	T	EE	A	D	L
**JX1602**	N	I	T	R	GKR	Y	-	A	T	EE	A	D	L
**GZ1601**	T	I	T	Q	RRG	H	-	T	T	DD	V	D	L
**HLJ1603-2**	T	I	T	Q	RRG	H	-	T	T	DD	V	D	L
**HLJ1603-1**	T	I	T	Q	RRG	H	-	T	T	DD	V	D	L
**HE1511**	T	I	T	Q	RRG	H	-	T	T	DD	V	D	L
**HLJ1506-2**	N	I	T	R	GKR	Y	-	A	T	EE	A	D	L
**GS1512**	N	I	T	R	GKR	Y	-	A	T	EE	A	D	L
**HLJ1508**	N	I	T	Q	RRG	H	-	A	T	DD	V	D	L
**G13**	N	I	T	R	GKR	Y	-	A	T	EE	A	D	L
**JL1511**	N	I	T	Q	RRG	H	-	A	T	DD	V	D	L
**HM590588**	T	I	T	Q	RRG	H	-	T	T	DD	V	D	L
**JQ690763**	N	I	T	Q	RRG	H	-	A	T	DD	V	D	L
**RS/BR/15/2S**	T	I	T	Q	RRG	H	-	T	T	DD	V	D	L
**HLJ1506-1**	T	I	T	Q	RRG	H	-	T	T	DD	V	D	L
**915F06007FD**	N	I	T	R	GKR	Y	-	A	T	EE	A	D	L
**G17**	T	I	T	R	RRG	Y	S	A	S	EE	A	D	L
**CL33**	N	I	T	R	GKR	Y	-	A	T	EE	A	D	L
**S53/It**	N	I	T	R	GKR	Y	-	A	T	EE	A	D	L
**HLJ1510**	N	I	T	R	GKR	Y	-	A	T	EE	A	D	L

^a^ Mutations were displayed in gray.

**Table 5:** Mutations at different amino acid sites in AGV2 VP3.

Strains	Substitution of Amino Acid Residues																				
	7	9	12	14	28	54	65	69	71	79	81	83	85	99	101	103	104	115	120	122	124
**HB2018S1**	R	R	T	Q	S	Y	A	D	G	V	L	Y ^a^	R	A	K	Q	Q	N	K	-	L
**NX1510**	R	R	T	Q	S	Y	A	D	G	V	L	H	R	A	K	Q	Q	N	K	-	L
**NX1506-2**	R	R	I	Q	S	Y	A	D	G	V	L	H	R	A	K	Q	Q	N	K	-	L
**NX1506-1**	R	R	T	Q	S	Y	A	D	G	V	L	H	R	A	K	Q	Q	N	K	-	L
**JL1508**	R	R	T	Q	S	Y	A	D	G	V	L	H	R	A	K	Q	Q	N	K	-	L
**LN1511**	R	R	T	Q	S	Y	A	D	G	V	L	H	R	A	K	Q	Q	N	K	-	L
**JX1602**	R	R	T	Q	S	Y	A	D	G	V	L	H	R	A	K	Q	Q	N	K	-	L
**GZ1601**	R	Q	T	R	C	Y	A	D	G	A	S	H	R	S	K	R	R	E	K	-	L
**HLJ1603-2**	R	Q	T	R	C	Y	A	D	G	A	S	H	R	S	K	R	R	E	K	-	L
**HLJ1603-1**	R	Q	T	R	C	Y	A	D	G	A	S	H	R	S	K	R	R	E	K	-	L
**HE1511**	R	Q	T	R	C	Y	A	D	G	A	S	H	R	S	K	R	R	E	K	-	L
**HLJ1506-2**	R	R	T	Q	S	Y	A	D	G	V	L	H	R	A	K	Q	Q	N	K	-	L
**GS1512**	R	R	T	Q	S	Y	A	D	G	V	L	H	R	A	K	Q	Q	N	K	-	L
**HLJ1508**	R	R	T	Q	C	S	V	A	E	V	L	H	R	S	R	Q	R	E	K	-	L
**G13**	R	R	T	Q	S	Y	A	D	G	V	L	H	R	A	K	Q	Q	N	K	-	L
**JL1511**	R	R	T	Q	C	S	V	A	E	V	L	H	R	S	R	Q	R	E	K	-	L
**HM590588**	R	Q	T	R	C	Y	A	D	G	A	S	H	R	S	K	Q	R	E	K	-	L
**JQ690763**	R	Q	T	R	C	Y	A	D	G	V	L	H	R	S	K	Q	R	E	K	-	L
**RS/BR/15/2S**	R	Q	T	R	C	Y	A	D	G	A	S	H	R	S	K	Q	R	E	K	-	L
**HLJ1506-1**	R	Q	T	R	C	Y	A	D	G	A	S	H	R	S	K	Q	R	E	K	-	L
**915F06007FD**	R	R	T	Q	S	Y	A	D	G	V	L	H	R	A	K	Q	Q	N	K	-	L
**G17**	H	Q	T	R	S	Y	A	D	G	V	L	H	K	A	K	Q	R	E	R	R	V
**CL33**	R	R	T	Q	S	Y	A	D	G	V	L	H	R	A	K	Q	Q	N	K	-	L
**S53/It**	R	R	T	Q	S	Y	A	D	G	V	L	H	R	A	K	Q	Q	N	K	-	L
**HLJ1510**	R	R	T	Q	S	Y	A	D	G	V	L	H	R	A	K	Q	Q	N	K	-	L

^a^ Mutations were displayed in gray.

## Supplementary Materials

The following are available online at https://www.mdpi.com/2076-0817/8/4/185/s1, Table S1: The sequence information of reference strains used in this study, Figure S1: Detection results of AGV2 in Farm B samples by electrophoresis.

## References

[1] Rosario K., Breitbart M., Harrach B., Segalés J., Delwart E., Biagini P., Varsani A. (2017). Revisiting the taxonomy of the family Circoviridae: Establishment of the genus Cyclovirus and removal of the genus Gyrovirus. Arch. Virol..

[2] Schat K.A. (2009). Chicken anemia virus. Curr. Top. Microbiol. Immunol..

[3] Rijsewijk F.A., Dos S.H.F., Teixeira T.F., Cibulski S.P., Varela A.P., Dezen D., Franco A.C., Roehe P.M. (2011). Discovery of a genome of a distant relative of chicken anemia virus reveals a new member of the genus Gyrovirus. Arch. Virol..

[4] Yao S., Tuo T., Gao X., Han C., Li Y., Gao Y., Zhang Y., Liu C., Qi X., Gao H. (2016). Avian gyrovirus 2 in poultry, China, 2015–2016. Emerg. Microbes Infect..

[5] Dos S.H.F., Knak M.B., de Castro F.L., Slongo J., Ritterbusch G.A., Klein T.A., Esteves P.A., Silva A.D., Trevisol I.M., Claassen E.A. (2012). Variants of the recently discovered avian gyrovirus 2 are detected in Southern Brazil and The Netherlands. Vet. Microbiol..

[6] Varela A.P., Dos S.H.F., Cibulski S.P., Scheffer C.M., Schmidt C., Sales L.F.E., Silva A.D., Esteves P.A., Franco A.C., Roehe P.M. (2014). Chicken anemia virus and avian gyrovirus 2 as contaminants in poultry vaccines. Biologicals.

[7] Maggi F., Macera L., Focosi D., Vatteroni M.L., Boggi U., Antonelli G., Eloit M., Pistello M. (2012). Human gyrovirus DNA in human blood, Italy. Emerg. Infect. Dis..

[8] Phan T.G., Li L., O’Ryan M.G., Cortes H., Mamani N., Bonkoungou I.J., Wang C., Leutenegger C.M., Delwart E. (2012). A third gyrovirus species in human faeces. J. Gen. Virol..

[9] Abolnik C., Wandrag D.B. (2014). Avian gyrovirus 2 and avirulent Newcastle disease virus coinfection in a chicken flock with neurologic symptoms and high mortalities. Avian Dis..

[10] Feher E., Pazar P., Kovacs E., Farkas S.L., Lengyel G., Jakab F., Martella V., Banyai K. (2014). Molecular detection and characterization of human gyroviruses identified in the ferret fecal virome. Arch. Virol..

[11] Sauvage V., Cheval J., Foulongne V., Gouilh M.A., Pariente K., Manuguerra J.C., Richardson J., Dereure O., Lecuit M., Burguiere A. (2011). Identification of the first human gyrovirus, a virus related to chicken anemia virus. J. Virol..

[12] Ye J., Tian X., Xie Q., Zhang Y., Sheng Y., Zhang Z., Wang C., Zhu H., Wang Y., Shao H. (2015). Avian Gyrovirus 2 DNA in fowl from live poultry markets and in healthy humans, China. Emerg. Infect. Dis..

[13] He Z., Zhang H., Gao S., Lercher M.J., Chen W.H., Hu S. (2016). Evolview v2: An online visualization and management tool for customized and annotated phylogenetic trees. Nucleic Acids Res..

[14] Martin D.P., Murrell B., Golden M., Khoosal A., Muhire B. (2015). RDP4: Detection and analysis of recombination patterns in virus genomes. Virus Evol..

[15] Noteborn M.H., de Boer G.F., van Roozelaar D.J., Karreman C., Kranenburg O., Vos J.G., Jeurissen S.H., Hoeben R.C., Zantema A., Koch G. (1991). Characterization of cloned chicken anemia virus DNA that contains all elements for the infectious replication cycle. J. Virol..

[16] Phenix K.V., Meehan B.M., Todd D., McNulty M.S. (1994). Transcriptional analysis and genome expression of chicken anaemia virus. J. Gen. Virol..

[17] Peng Y.H., Nishizawa T., Takahashi M., Ishikawa T., Yoshikawa A., Okamoto H. (2002). Analysis of the entire genomes of thirteen TT virus variants classifiable into the fourth and fifth genetic groups, isolated from viremic infants. Arch. Virol..

[18] Peters M.A., Jackson D.C., Crabb B.S., Browning G.F. (2002). Chicken anemia virus VP2 is a novel dual specificity protein phosphatase. J. Biol. Chem..

[19] Takahashi K., Iwasa Y., Hijikata M., Mishiro S. (2000). Identification of a new human DNA virus (TTV-like mini virus, TLMV) intermediately related to TT virus and chicken anemia virus. Arch. Virol..

[20] Noteborn M.H., Verschueren C.A., Koch G., Van der Eb A.J. (1998). Simultaneous expression of recombinant baculovirus-encoded chicken anaemia virus (CAV) proteins VP1 and VP2 is required for formation of the CAV-specific neutralizing epitope. J. Gen. Virol..

[21] Cheng J.H., Sheu S.C., Lien Y.Y., Lee M.S., Chen H.J., Su W.H., Lee M.S. (2012). Identification of the NLS and NES motifs of VP2 from chicken anemia virus and the interaction of VP2 with mini-chromosome maintenance protein 3. BMC Vet. Res..

[22] Taebunpakul P., Sayan B.S., Flinterman M., Klanrit P., Gaken J., Odell E.W., Melino G., Tavassoli M. (2012). Apoptin induces apoptosis by changing the equilibrium between the stability of TAp73 and DeltaNp73 isoforms through ubiquitin ligase PIR2. Apoptosis.

