# Supplementary material for: Genetic Analysis of Avian Gyrovirus 2 Variant-Related Gyrovirus Detected in Farmed King Ratsnake (Elaphe carinata): The First Report from China
Source: Pathogens. 2019 Oct 12;8(4):185. doi: 10.3390/pathogens8040185 (PMC6963503; doi:10.3390/pathogens8040185)
Supplement: Supplementary file 1 [file pathogens-08-00185-s001.pdf]

Supplement 1. The sequence information of reference strains used in this study

Strains	Sample	Source	Date	Country	Accession Nos.
**NX1506-1**	Liver and spleen	*Gallus gallus*	25.06.2015	China	KX708508
**NX1506-2**	Liver and spleen	*Gallus gallus*	26.06.2015	China	KX708509
**NX1510**	Liver and spleen	*Gallus gallus*	27.10.2015	China	KX708513
**LN1511**	Liver and spleen	*Gallus gallus*	27.11.2015	China	KX708515
**JL1508**	Liver and spleen	*Gallus gallus*	25.08.2015	China	KX708511
**JX1602**	Liver and spleen	*Gallus gallus*	22.02.2016	China	KX708519
**CL33**	Feces	*Homo sapiens*	2008	Chile	JQ308212
**G13**	Feces	*Mustela putorius furo*	2011	Hungary	KJ452214
**S53-It**	Serum	*Gallus gallus*	12.01.2014	Italy	KU168250
**HLJ1506-2**	Liver and spleen	*Gallus gallus*	23.06.2015	China	KX708522
**GS1512**	Liver and spleen	*Gallus gallus*	07.12.2015	China	KX708517
**HLJ1510**	Liver and spleen	*Gallus gallus*	20.10.2015	China	KX708507
**915F06007FD**	Healthy face skin	*Homo sapiens*	05.2009	France	FR823283
**GZ1601**	Liver and spleen	*Gallus gallus*	04.01.2016	China	KX708518
**Ave3**	Serum	*Gallus gallus*	14.07.2016	Brazil	HM590588
**HE1511**	Liver and spleen	*Gallus gallus*	20.11.2016	China	KX708514
**HLJ1603-2**	Liver and spleen	*Gallus gallus*	03.03.2016	China	KX708521
**HLJ1603-1**	Liver and spleen	*Gallus gallus*	03.03.2016	China	KX708520
**HLJ1508**	Liver and spleen	*Gallus gallus*	03.08.2016	China	KX708510
**JL1511**	Liver and spleen	*Gallus gallus*	27.11.2015	China	KX708516
**HLJ1506-1**	Liver and spleen	*Gallus gallus*	03.06.2016	China	KX708506
**G17**	Feces	*Mustela putorius furo*	2011	Hungary	KJ452213
**RS-BR-15-2S**	Feces	*Gallus gallus*	2015	Brazil	MG846492

**RS-BR-2015**	Feces	*Gallus gallus*	31.07.2015	Brazil	KY039279
**JQ690763.1**	Feces	*Homo sapiens*	2012	China	JQ690763

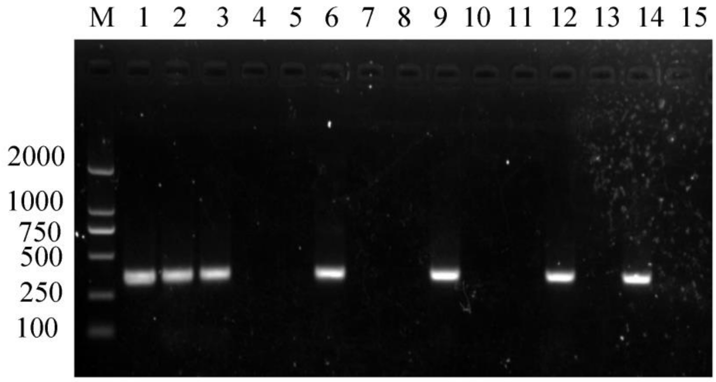

Supplement 2. Detection results of AGV2 in Farm B samples by electrophoresis. M: 2000 marker; 1–11: fecal samples, 12 and 13: liver tissue samples; 14: positive control; 15: negative control. The DNA template used in the positive control was the preserved and correctly sequenced AGV2 sample and that in the negative control was sterile water.
